# Measures of physical fitness and postoperative mortality in patients with colorectal cancer: national cohort study

**DOI:** 10.1093/bjsopen/zrag130

**Published:** 2026-10-23

**Authors:** Adil Rashid, Helen A Blake, Leo Watton, Nicola S Fearnhead, Lu Han, Jan van der Meulen, Kate Walker

**Affiliations:** Department of Health Services Research and Policy, London School of Hygiene and Tropical Medicine, London, UK; Clinical Effectiveness Unit, Royal College of Surgeons of England, London, UK; Research Department of Primary Care and Population Health, UCL Institute of Epidemiology and Health Care, London, UK; Clinical Effectiveness Unit, Royal College of Surgeons of England, London, UK; Department of Colorectal Surgery, Warrington and Halton NHS Trust, Warrington, UK; Institute of Science of Integrative Molecular Biology, University of Liverpool, Liverpool, UK; Department of Colorectal Surgery, Cambridge University Hospital NHS Foundation Trust, Cambridge, UK; Department of Health Services Research and Policy, London School of Hygiene and Tropical Medicine, London, UK; Clinical Effectiveness Unit, Royal College of Surgeons of England, London, UK; Department of Health Services Research and Policy, London School of Hygiene and Tropical Medicine, London, UK; Clinical Effectiveness Unit, Royal College of Surgeons of England, London, UK; Department of Health Services Research and Policy, London School of Hygiene and Tropical Medicine, London, UK; Clinical Effectiveness Unit, Royal College of Surgeons of England, London, UK

**Keywords:** risk adjustment, quality assurance, ASA grade, colorectal surgery, performance status

## Abstract

**Background:**

Measures of physical fitness are essential for case mix adjustment when comparing surgical outcomes between patient groups, surgical approaches, and healthcare providers. Commonly used measures include clinician-reported American Society of Anesthesiologists (ASA) grade and Eastern Cooperative Oncology Group (ECOG) performance status, as well as the Secondary Care Administrative Records Frailty (SCARF) index, derived from routinely collected administrative hospital data. This study compared how these measures of physical fitness, perform for case mix adjustment of postoperative colorectal cancer mortality.

**Methods:**

All patients diagnosed with colorectal cancer (CRC) between April 2017 and March 2022 in the English and Welsh National Health Service who underwent surgery were included. The primary outcome was 90-day postoperative mortality. Using an established risk-adjustment model, different combinations of ASA grade, performance status, and the SCARF index were added to create eight models for 90-day mortality risk adjustment. Model performance was compared with a full model including all three measures of physical fitness. The basic model included no measures of physical fitness. The scaled Brier score (SBS) measured overall model performance and the C-statistic measured discriminatory ability.

**Results:**

Among 91 957 patients, 90-day mortality was 2.8%. A model including both ASA grade and performance status achieved equivalent performance (SBS 0.092; C-statistic 0.833 (95% confidence interval (c.i.) 0.825 to 0.841)) to the full model (SBS 0.092; C-statistic 0.834 (95% c.i. 0.827 to 0.842)). The basic model with only performance status was slightly inferior (SBS 0.076; C-statistic 0.822 (95% c.i. 0.814 to 0.830)) to the basic model with only ASA grade (SBS 0.084; C-statistic 0.827 (95% c.i. 0.819 to 0.835)).

**Conclusion:**

Performance status and ASA grade together accurately adjust for physical fitness when risk adjusting postoperative mortality in patients with CRC. When only one measure is available, performance status or ASA grade alone is suitable to support risk-adjusted hospital benchmarking.

## Teaser

This study compared how different measures of physical fitness, namely American Society of Anesthesiologists (ASA) grade, performance status, and the Secondary Care Administrative Records Frailty (SCARF) index, perform for risk adjustment of postoperative colorectal cancer mortality. Performance status and ASA grade together accurately adjust for physical fitness. Where only one measure is available, performance status or ASA grade alone is suitable for hospital benchmarking.

## Introduction

There is growing emphasis on outcome-reporting programmes to evaluate perioperative care and surgical outcomes for patients with colorectal cancer (CRC)^1^. These programmes, including national audits and registries, have highlighted variations across hospitals, including in access to timely surgery, the use of evidence-based care, and postoperative morbidity and mortality^2,3^.

The comparison of surgical outcomes across hospitals is increasingly being used to improve the safety and quality of healthcare as part of quality assurance^4,5^. It allows poorly performing hospitals to be identified, and best practices at high-performing hospitals to be disseminated^6^. Comparisons of performance among hospitals requires robust adjustment for differences in risk to ensure that institutions that treat higher-risk patients are not unfairly penalized. Quality measures and mortality data are often publicly reported^7^, and accurate risk adjustment reduces any inclination for clinical teams to avoid treating high-risk patients^8^. More broadly, robust risk adjustment is essential in observational studies of surgical outcomes, where differences in baseline patient characteristics may confound comparisons between surgical approaches or care pathways.

Measures of physical fitness are essential for case mix adjustment. Physical fitness influences patient selection for surgical resection^9,10^, recommendations on anastomosis *versus* stoma, and postoperative outcomes^11,12^. Measures commonly used in existing risk adjustment models include the American Society of Anesthesiologists (ASA) grade^13–16^, Eastern Cooperative Oncology Group (ECOG) performance status^17^, and the Secondary Care Administrative Records Frailty (SCARF) index^18,19^.

ASA grade is routinely recorded by anaesthetists before surgery and provides a global assessment of the severity of systemic disease^13–15^. Performance status categorizes patients according to their ability to perform daily activities, ranging from fully active to completely disabled^17^. Performance status reflects a patient’s functional capacity. Both ASA grade and performance status rely on clinical judgment and require bespoke data collection, which can limit their completeness for large-scale outcome reporting^20^. In contrast, the SCARF index is derived entirely from routinely collected administrative records in Hospital Episode Statistics (HES), without the need for additional data collection. The SCARF index captures 32 potential deficits across functional impairment, conditions that predominantly affect older patients, problems with nutrition, cognition, mood, and medical co-morbidities^18^. The SCARF index is based on the cumulative deficit model of frailty, which conceptualizes frailty as the accumulation of deficits across multiple physiological systems, presenting as clinical signs or symptoms, physical or cognitive impairments, or diseases^21^. Each of these measures captures distinct domains related to physical fitness and vulnerability to stressors. These domains are conceptually distinct, with systemic disease, functional capacity, and frailty representing related aspects of patient fitness^22,23^.

Using national population data, this study aimed to compare how these domains of physical fitness, namely ASA grade, performance status, and the SCARF index, perform within an established risk-adjustment model for postoperative CRC mortality^14^, accounting for the availability and completeness of each measure. This comparison will inform the selection of physical fitness measures for future risk models in CRC, supporting equitable hospital benchmarking of outcomes.

## Methods

### Data sources

This population-based cohort study used data from the National Bowel Cancer Audit (NBOCA)^24^, HES^25^, Patient Episode Database for Wales (PEDW)^26^, and Office for National Statistics (ONS) mortality data set^27^, linked at the patient level.

The NBOCA is a prospective mandatory database for all patients newly diagnosed with CRC in the English and Welsh National Health Service (NHS). Data items extracted include age, sex, date of diagnosis, date of surgical resection (defined as removal of part or all of the colon and/or rectum), tumour site, clinical (c) and pathological (p) stage according to the tumour node and metastasis (TNM) staging system, ASA grade, performance status, and mode of admission.

The HES and PEDW are national administrative data sets of all admissions to English and Welsh NHS hospitals, respectively. The HES and PEDW provide information on sex, mode of admission (elective or emergency), and International Classification of Diseases, Tenth Revision (ICD-10) codes^28^ for acute and chronic medical conditions.

### Study population

The study included all patients diagnosed with primary CRC between 1 April 2017 and 31 March 2022 who subsequently underwent surgical resection for CRC, as defined by the NBOCA^29^ using the Classification of Interventions and Procedures (OPCS-4) codes listed in *Table S1*. ONS mortality data, to obtain 90-day mortality, were available until 31 March 2023. Patients who could not be linked to HES, PEDW, and ONS mortality data, or whose date of death was before the date of surgery, were excluded.

### Definition of variables

*Table S2* provides sources of information for each of the variables included in the analysis.

For patients with missing mode of admission data in the HES, the NBOCA source of referral was used as a substitute. Patient diagnosis codes were identified using HES and PEDW ICD-10 codes recorded in any hospital admission within the 2 years preceding surgical resection, including the index surgical admission. These diagnosis codes were then used to calculate the Royal College of Surgeons (RCS) Charlson score, excluding malignancy^30^, and the SCARF index^18^.

The SCARF index was originally developed to be calculated at the time of a cancer diagnosis using a 2-year look-back period. When calculated at the time of surgical resection, ICD-10 diagnosis codes recorded during the index surgical admission may represent acute postoperative complications rather than pre-existing co-morbidities. To avoid misclassifying postoperative complications as pre-existing co-morbidities, the SCARF index was modified in keeping with the RCS Charlson score methodology by including only ICD-10 codes that represent acute postoperative complications in hospital admissions before the surgical admission^30^. ICD-10 codes that could represent acute postoperative complications were identified in a clinical consensus exercise among the authors (*Table S3*), applying the following principles:

- ICD-10 codes already classified as acute in the RCS Charlson score^30^ were classified as acute in the SCARF index.
- ICD-10 codes were only reviewed if they occurred in ≥ 0.01% of index surgical admissions.
- ICD-10 codes describing events with a sudden onset that are clinically plausible complications or consequences of CRC resection were classified as acute.

ICD-10 codes in the RCS Charlson score were also re-reviewed using the above principles (*Table S4*).

The RCS Charlson score and SCARF index were recorded as missing for patients without an index surgical admission in the HES or PEDW, even though surgical resection was recorded in the NBOCA.

### Outcome of interest

The primary outcome of the risk-adjustment model was all-cause 90-day mortality. All patients either experienced the outcome or had at least 90 days of postoperative follow-up.

### Statistical analysis

The basic risk-adjustment model included demographic characteristics (age and sex), tumour characteristics (cancer site; T, N, and M categories), co-morbidity burden (RCS Charlson score^30^), and health service factors (mode of admission). The model incorporates age as a linear plus quadratic term and includes an interaction between age and metastatic disease. This basic model plus the ASA grade has previously been validated and used in public outcome reporting in England and Wales^14,31^.

Building on this framework, eight risk-adjustment models were created, including variables presented in *Table 1*. The full model included all three measures of physical fitness (ASA grade, performance status, and SCARF index) in addition to the demographic, tumour, co-morbidity, and health service variables in the basic model. Six additional models evaluated different combinations of ASA grade, performance status, and SCARF index in addition to the variables in the basic model. This approach enabled the assessment of both individual and synergistic contributions of each physical fitness measure.

**Table 1:** Patient and tumour characteristics included in each model

Model	Variables
Basic model	Age, sex, year of diagnosis, cancer site, TNM T category, TNM N category, TNM M category, mode of admission, RCS Charlson score
ASA grade	Age, sex, year of diagnosis, cancer site, TNM T category, TNM N category, TNM M category, mode of admission, RCS Charlson score, ASA grade
Performance status	Age, sex, year of diagnosis, cancer site, TNM T category, TNM N category, TNM M category, mode of admission, RCS Charlson score, performance status
SCARF index	Age, sex, year of diagnosis, cancer site, TNM T category, TNM N category, TNM M category, mode of admission, RCS Charlson score, SCARF index
ASA grade and performance status	Age, sex, year of diagnosis, cancer site, TNM T category, TNM N category, TNM M category, mode of admission, RCS Charlson score, ASA grade, performance status
ASA grade and SCARF index	Age, sex, year of diagnosis, cancer site, TNM T category, TNM N category, TNM M category, mode of admission, RCS Charlson score, ASA grade, SCARF index
SCARF index and performance status	Age, sex, year of diagnosis, cancer site, TNM T category, TNM N category, TNM M category, mode of admission, RCS Charlson score, performance status, SCARF index
Full model	Age, sex, year of diagnosis, cancer site, TNM T category, TNM N category, TNM M category, mode of admission, RCS Charlson score, ASA grade, performance status, SCARF index

TNM, tumour node metastasis staging system; RCS, Royal College of Surgeons; ASA, American Society of Anesthesiologists; SCARF, Secondary Care Administrative Records Frailty.

Multiple imputation with chained equations was used to handle missing data, assuming missingness is conditional on 90-day mortality and other observed information^32^. Overall, 19.5% of patients had missing data for at least one variable in the full model, so 20 imputations were created. Data on 90-day mortality, age, sex, year of diagnosis, tumour site, and procedure type were complete for all patients. Binary variables were imputed using logistic regression; categorical variables and continuous variables were imputed using predictive mean matching with values drawn at random from a pool of *k* = 5 observations with similar predicted values^33^.

For each of the eight models (*Table 1*), the McFadden pseudo-*R*^2^ was used as a measure of goodness-of-fit^34^. This statistic compares the maximized log-likelihood of the fitted model with that of a null model (a model with no predictors) and indicates how much better the model fits the observed data than a model with no predictors. Each model’s pseudo-*R*^2^ was compared to that of the full model. Other performance measures calculated were the C-statistic and the scaled Brier score (SBS). The C-statistic quantifies the model's discrimination, ranging from 0.5 (non-informative) to 1 (perfect discrimination)^35–38^. Discrimination refers to the model’s ability to distinguish between individuals who do and do not experience the event. The SBS quantifies the average prediction error (accuracy) of the model predictions compared with a naïve non-informative model. The calculated SBS ranged from 0 (non-informative) to 1 (perfect predictions)^35,39,40^. Pseudo-*R*^2^, C-statistic, and SBS values were pooled over the imputed data sets using Rubin’s rules^41,42^.

The calibration of each model was checked by plotting observed *versus* predicted mortality by deciles of predicted risk, averaged over all imputed data sets. Calibration measures how accurately the model’s predictions match the observed outcomes. Because the χ^2^ statistic from Hosmer–Lemeshow tests cannot be pooled using Rubin’s rules, an F-statistic was calculated to account for between-imputation variation^43^. With very large data sets Hosmer–Lemeshow tests are highly sensitive to small unimportant differences in observed and predicted risk. Calibration was considered to be a problem only if there was statistical evidence of a lack of fit from the F-statistic of the combined Hosmer–Lemeshow tests, and the size of differences between observed and predicted risk in deciles of risk was large enough to be clinically important.

STATA^®^ version 17 (StataCorp, College Station, TX, USA) was used for all analyses. This work has also been reported in line with the strengthening the reporting of observational studies in epidemiology (STROBE) statement^44^ (*Table S5*).

## Results

### Study population

In all, 92 397 patients were diagnosed with CRC between 1 April 2017 and 31 March 2022 and underwent surgical resection. Of the included patients, 91 905 (99.5%) were linked to HES or PEDW and the ONS mortality data set. After excluding 27 patients with a recorded date of death before surgery, 91 878 patients were included in the final cohort for risk adjustment for physical fitness (*Fig. 1*).

**Figure 1:**
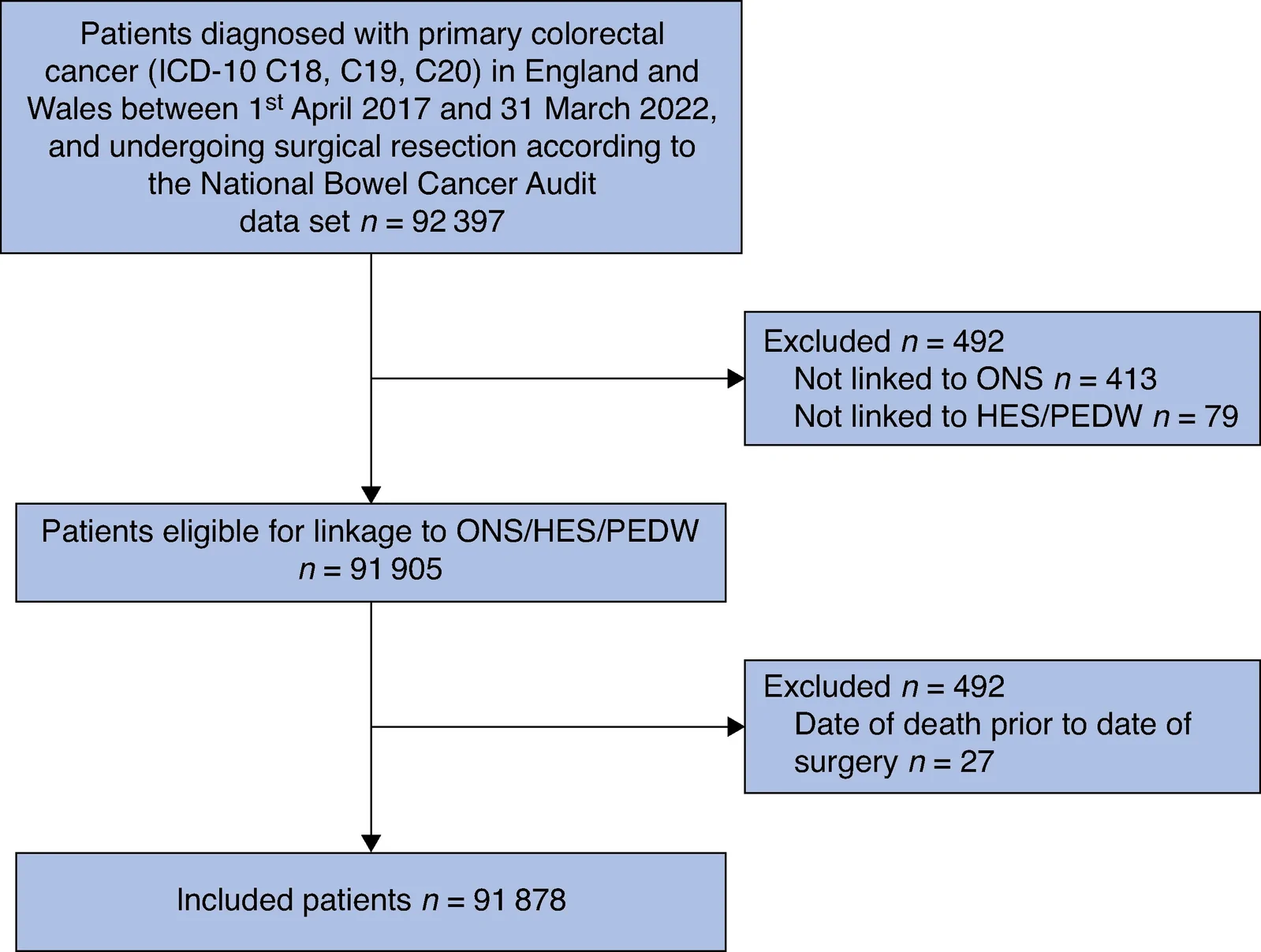
Flow chart of patient inclusion Among 92 397 patients diagnosed with primary colorectal cancer in England and Wales between 1 April 2017 and 31 March 2022 who underwent surgical resection, 413 were not linked to the ONS mortality data set and 79 were not linked to the HES or PEDW. This left 91 905 eligible patients. A further 27 patients were excluded because their recorded date of death preceded the date of surgery, resulting in a final cohort of 91 878 patients. ONS, Office for National Statistics; HES, Hospital Episode Statistics; PEDW, Patient Episode Database for Wales; ICD-10, International Classification of Diseases, Tenth Revision.

The mean(standard deviation) age of the included patients was 68.8(11.7) years, 56.3% were male, and 6.4% had metastatic CRC (stage IV disease). Patient and tumour characteristics and 90-day mortality are summarized in *Table 2*. Missing data for performance status was the highest, at 10.6%. This compares with 5.1% missing data for ASA grade and 5.3% for the SCARF index and RCS Charlson score. Missing data for all other variables was < 2%.

**Table 2:** Distribution of patient and tumour characteristics and 90-day mortality

	Total cohort	90-day mortality (%)
Total	91 878 (100.0%)	2609 (2.8%)
**Patient and tumour characteristics**		
Age (years)		
< 50	5989 (6.5%)	69 (1.2%)
50–59	11 961 (13.0%)	155 (1.3%)
60–74	42 741 (46.5%)	886 (2.1%)
75–84	25 510 (27.8%)	1074 (4.2%)
≥ 85	5677 (6.2%)	425 (7.5%)
Sex		
Male	51 687 (56.3%)	1530 (3.0%)
Female	40 191 (43.7%)	1079 (2.7%)
Cancer site		
Caecum/ascending colon	27 225 (29.6%)	992 (3.6%)
Hepatic flexure	4559 (5.0%)	179 (3.9%)
Transverse colon	6547 (7.1%)	266 (4.1%)
Splenic flexure/descending	5902 (6.4%)	225 (3.8%)
Sigmoid colon	21 519 (23.4%)	515 (2.4%)
Rectosigmoid	4860 (5.3%)	105 (2.2%)
Rectal	21 266 (23.2%)	327 (1.5%)
T category		
T1	8163 (8.9%)	84 (1.0%)
T2	15 677 (17.1%)	240 (1.5%)
T3	46 279 (50.4%)	960 (2.1%)
T4	20 927 (22.8%)	1287 (6.1%)
Missing	832 (0.9%)	38 (4.6%)
N category		
N0	52 911 (57.6%)	1142 (2.2%)
N1	25 558 (27.8%)	694 (2.7%)
N2	12 633 (13.8%)	737 (5.8%)
Missing	776 (0.8%)	36 (4.6%)
M category		
M0	84 793 (92.3%)	2028 (2.4%)
M1	5854 (6.4%)	522 (8.9%)
Missing	1231 (1.3%)	59 (4.8%)
Mode of admission		
Elective	77 236 (84.1%)	1263 (1.6%)
Emergency	13 768 (15.0%)	1315 (9.6%)
Missing	874 (1.0%)	31 (3.5%)
Year of diagnosis		
2017–2018	19 038 (20.7%)	597 (3.1%)
2018–2019	17 910 (19.5%)	541 (3.0%)
2019–2020	19 419 (21.1%)	505 (2.6%)
2020–2021	16 551 (18.0%)	502 (3.0%)
2021–2022	18 960 (20.6%)	464 (2.4%)
RCS Charlson score		
0	43 617 (47.5%)	715 (1.6%)
1	26 815 (29.2%)	788 (2.9%)
2+	16 595 (18.1%)	895 (5.4%)
Missing	4851 (5.3%)	211 (4.3%)
ASA grade		
I	8728 (9.5%)	71 (0.8%)
II	48 531 (52.8%)	650 (1.3%)
III	27 680 (30.1%)	1252 (4.5%)
IV or V	2281 (2.5%)	451 (19.8%)
Missing	4658 (5.1%)	185 (4.0%)
Frailty (SCARF index)		
Fit	22 647 (24.7%)	298 (1.3%)
Mild frailty	29 190 (31.8%)	647 (2.2%)
Moderate frailty	24 361 (26.5%)	823 (3.4%)
Severe frailty	10 829 (11.8%)	630 (5.8%)
Missing	4851 (5.3%)	211 (4.3%)
Performance status		
0	48 057 (52.3%)	631 (1.3%)
1	24 844 (27.0%)	701 (2.8%)
2	7565 (8.2%)	505 (6.7%)
3+	1684 (1.8%)	260 (15.4%)
Missing	9728 (10.6%)	512 (5.3%)

Values are *n* (%). Percentages in the total cohort column, represent the proportion of the total study population; percentages in the 90-day mortality column, represent the mortality rate within each patient and tumour characteristic subgroup. RCS, Royal College of Surgeons; ASA, American Society of Anesthesiologists; SCARF, Secondary Care Administrative Records Frailty.

### Factors associated with 90-day mortality

Ninety-day mortality increased with age (1.2% *versus* 7.5% for age < 50 *versus* ≥ 85 years, respectively), advanced disease (6.1% *versus* 1.0% for T4 *versus* T1, respectively; 8.9% *versus* 2.4% for M1 *versus* T0, respectively), and emergency admission (9.6% *versus* 1.6% for elective admission). Ninety-day mortality was 19.8% among patients with ASA grades IV–V, compared with 0.8% for patients with ASA grade I. Similar trends were observed for performance status (90-day mortality 1.3% *versus* 15.4% for 0 *versus* 3+, respectively) and SCARF index (1.3% *versus*: 5.8% for fit *versus* severe frailty, respectively). *Table 3* presents the adjusted odds ratios for 90-day mortality for each variable in the full model, with the effect of age reported separately for patients with and without metastases. The strongest predictors of 90-day mortality were ASA grade, performance status, and mode of admission.

**Table 3:** Adjusted odds ratios and 95% confidence intervals for the variables in the full model, using multiple imputation to handle missing data

	Adjusted odds ratio
Age* (years)	
No metastasis	
50	0.49 (0.41, 0.58)
60	0.70 (0.66, 0.75)
70	1
80	1.42 (1.34, 1.50)
90	2.00 (1.72, 2.33)
Metastasis	
50	0.93 (0.79, 1.10)
60	0.96 (0.89, 1.03)
70	1
80	1.06 (0.94, 1.20)
90	1.15 (0.84, 1.58)
**Sex**	
Male	1
Female	0.77 (0.71, 0.84)
**Cancer site**	
Caecum/ascending colon	1
Hepatic flexure	1.19 (1.00, 1.41)
Transverse colon	1.15 (0.99, 1.34)
Splenic flexure/descending colon	1.11 (0.95, 1.30)
Sigmoid colon	0.86 (0.77, 0.97)
Rectosigmoid	1.13 (0.91, 1.40)
Rectal	1.11 (0.96, 1.27)
**T category**	
T1	1
T2	1.26 (0.97, 1.62)
T3	1.21 (0.96, 1.53)
T4	1.87 (1.47, 2.38)
**N category**	
N0	1
N1	0.92 (0.83, 1.02)
N2	1.54 (1.37, 1.73)
**M category**	
M0	1
M1	2.17 (1.88, 2.50)
**Mode of admission**	
Elective	1
Emergency	3.16 (2.86, 3.49)
**Year of diagnosis**	
2017–2018	1
2018–2019	0.99 (0.87, 1.12)
2019–2020	0.88 (0.77, 1.00)
2020–2021	0.97 (0.86, 1.11)
2021–2022	0.87 (0.76, 0.99)
**RCS Charlson score**	
0	1
1	1.26 (1.12, 1.41)
2+	1.57 (1.37, 1.80)
**ASA grade**	
I	1
II	1.23 (0.94, 1.60)
III	2.25 (1.73, 2.93)
IV or V	5.66 (4.26, 7.53)
**Frailty (SCARF index)**	
Fit	1
Mild frailty	1.24 (1.07, 1.44)
Moderate frailty	1.31 (1.12, 1.55)
Severe frailty	1.47 (1.21, 1.78)
**Performance status**	
0	1
1	1.31 (1.17, 1.47)
2	2.04 (1.78, 2.33)
3+	3.07 (2.57, 3.67)

Values in parentheses are 95% confidence intervals. *Adjusted odds ratios are presented for select age years because age was modelled a continuous variable using a linear plus quadratic term, with the reference value being 70 years. RCS, Royal College of Surgeons; ASA, American Society of Anesthesiologists; SCARF, Secondary Care Administrative Records Frailty.

### Model performance for 90-day mortality

*Figure 2* shows model performance measures for each of the eight risk-adjustment models. The relative pseudo-*R*^2^ is shown in descending order. *Table 4* shows that the full model had the highest goodness of fit, with a pooled pseudo-*R*^2^ of 0.192. The basic model plus ASA grade and performance status achieved 99.6% of the full model’s pseudo-*R*^2^. When focusing on models containing only one measure of physical fitness, ASA grade had the highest goodness of fit, achieving 94.2% of the full model’s pseudo-*R*^2^. By comparison, the SCARF index had the lowest goodness-of-fit. The basic model plus SCARF index achieved 82.2% of the full model’s pseudo-*R*^2^, similar to the basic model alone, which achieved 80.2% of the full model’s value.

**Figure 2:**
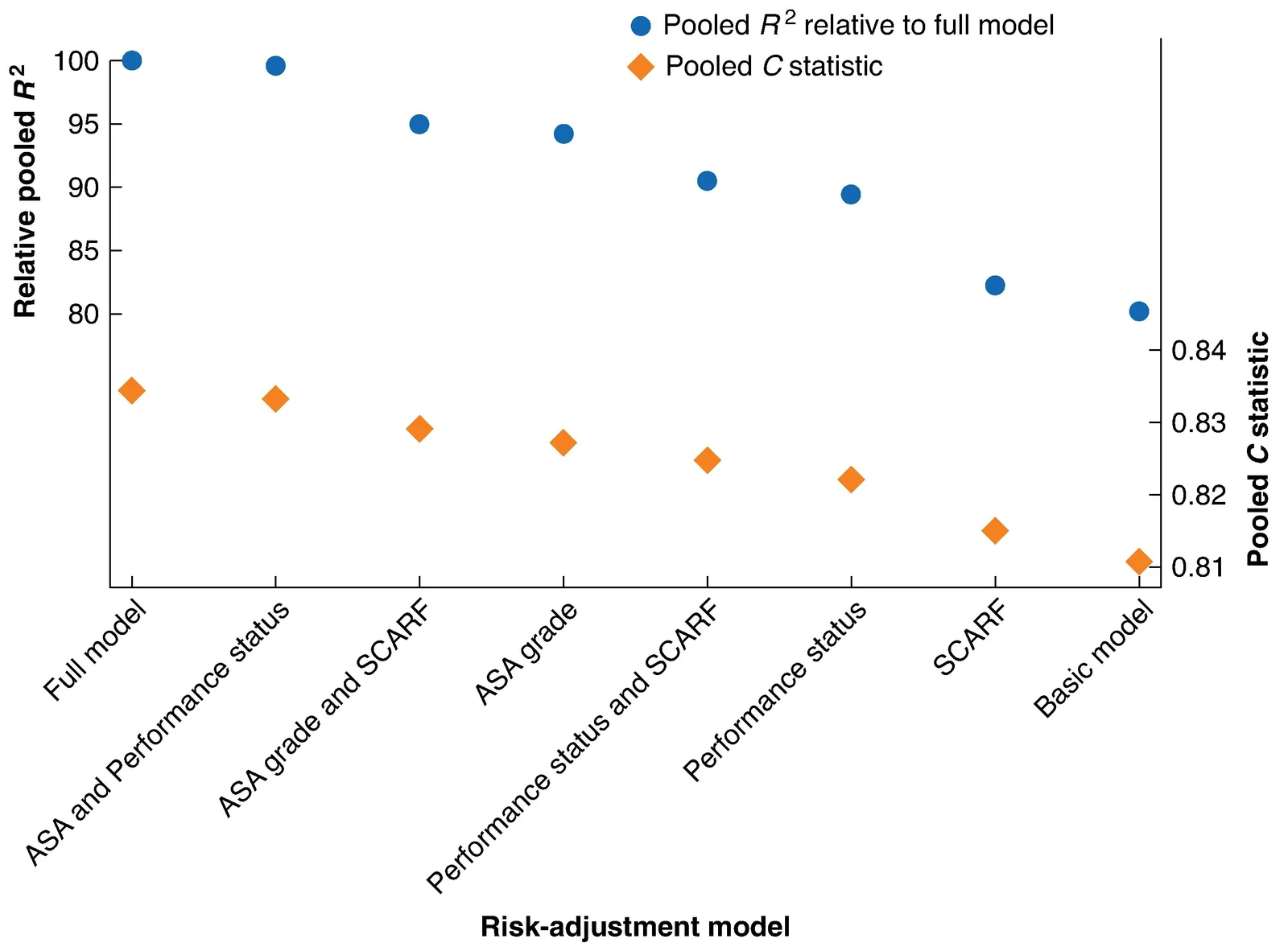
Pooled C-statistic and relative pooled pseudo-*R*^2^ for each of the eight risk-adjustment models for 90-day mortality Dot plots comparing the relative pooled pseudo-*R*^2^ and pooled C-statistic across the eight risk-adjustment models for 90-day mortality. ASA, American Society of Anesthesiologists; SCARF, Secondary Care Administrative Records Frailty.

**Table 4:** Model performance measures for each of the eight models, with relative pseudo-R-squared in descending order

Model	Pooled pseudo-*R*^2^	Pooled pseudo-*R*^2^ (% of full model)	Pooled C-statistic	Hosmer–Lemeshow *P*	Scaled Brier score
Full model	0.192	100.0%	0.834 (0.827, 0.842)	< 0.001	0.092
Basic model plus ASA grade and performance status	0.191	99.6%	0.833 (0.825, 0.841)	< 0.001	0.092
Basic model plus ASA grade and SCARF index	0.182	94.9%	0.829 (0.821, 0.837)	< 0.001	0.084
Basic model plus ASA grade	0.181	94.2%	0.827 (0.819, 0.835)	< 0.001	0.084
Basic model plus performance status and SCARF index	0.174	90.5%	0.825 (0.817, 0.833)	< 0.001	0.077
Basic model plus performance status	0.172	89.4%	0.822 (0.814, 0.830)	< 0.001	0.076
Basic model plus SCARF index	0.158	82.2%	0.815 (0.807, 0.823)	< 0.001	0.065
Basic model	0.154	80.2%	0.811 (0.803, 0.819)	< 0.001	0.063

Values in parentheses are 95% confidence intervals. ASA, American Society of Anesthesiologists; SCARF, Secondary Care Administrative Records Frailty.

Similar trends were observed when focusing on model discrimination. The full model had the highest C-statistic (0.834; 95% confidence interval (c.i.) 0.827 to 0.842). The basic model plus ASA grade and performance status had an equivalent C-statistic (0.833; 95% c.i. 0.825 to 0.841). When comparing models with a single measure of physical fitness, ASA grade had the highest C-statistic, at 0.827 (95% c.i. 0.819 to 0.835), and the SCARF index had the lowest, at 0.815 (95% c.i. 0.807 to 0.823), although their 95% confidence intervals overlapped.

The full model had the highest SBS (0.092), with equivalent predictive accuracy observed for the basic model plus ASA grade and performance status (0.092). Consistent with other performance measures, the SBS for the basic model plus the SCARF index (0.065) was similar to that of the basic model alone (0.063).

The basic model plus performance status achieved similar discrimination and calibration (SBS 0.076; C-statistic 0.822 (95% c.i. 0.814 to 0.830)) to the basic model plus ASA grade (SBS 0.084; C-statistic 0.827 (95% c.i. 0.819 to 0.835)).

Although the F-statistic of the combined Hosmer–Lemeshow tests gave statistical evidence of a lack of fit for all eight models (*P* < 0.001), the absolute difference between observed and predicted mortality was < 0.5% in each decile of predicted risk for all models except the basic model, the basic model plus the SCARF index, and the basic model plus ASA grade and SCARF index (*Fig. 3*).

**Figure 3:**
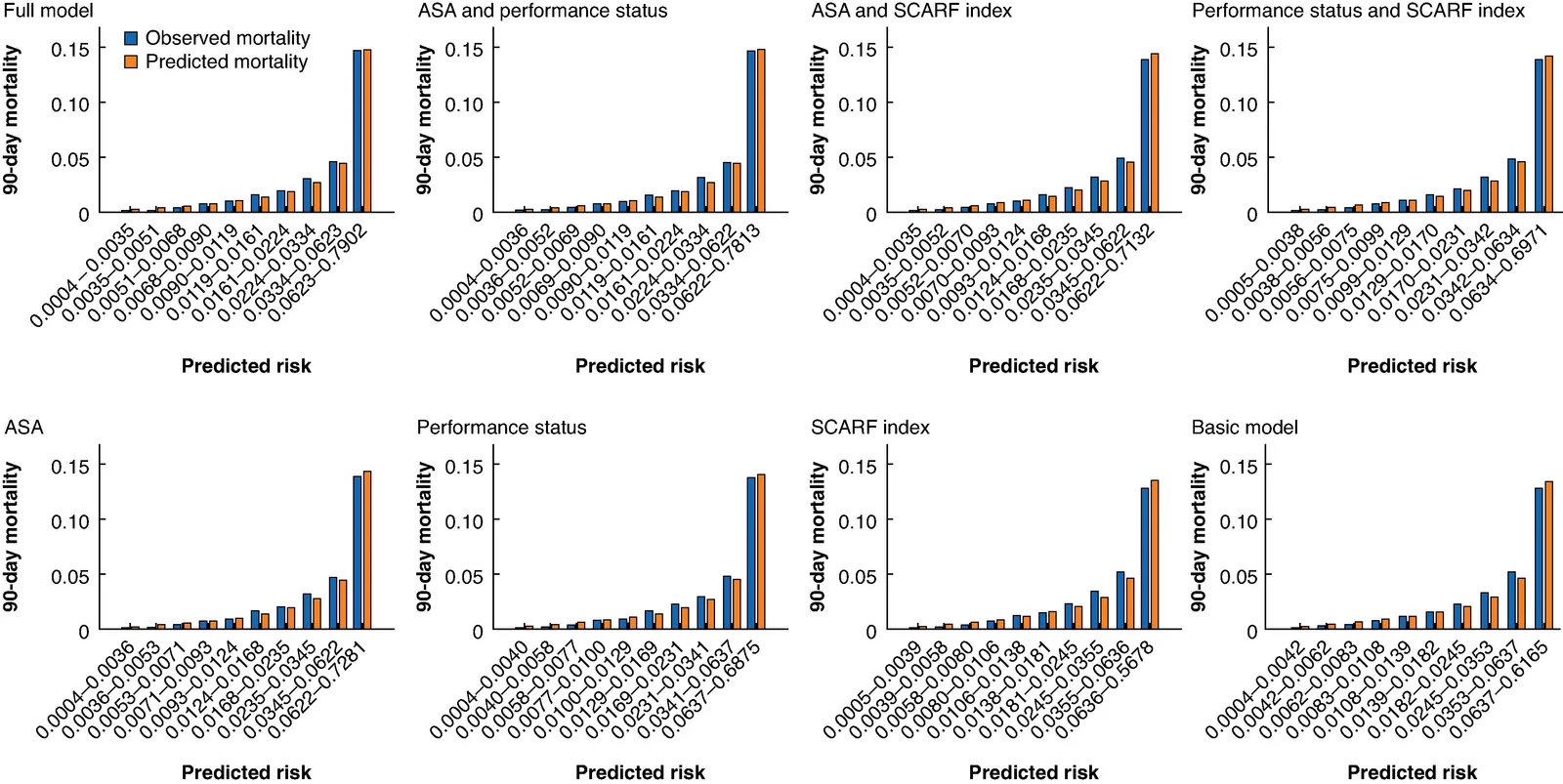
Calibration plots showing the proportion of patients who died within 90 days (observed mortality) *versus* mortality predicted for each of the eight models Calibration plots comparing observed and predicted 90-day mortality across deciles of predicted risk for each risk-adjustment model. The absolute difference between observed and predicted mortality was < 0.5% in each decile of predicted risk for all models except the basic model, the basic model plus the SCARF index and the basic model plus ASA grade and SCARF index. SCARF, Secondary Care Administrative Records Frailty; ASA, American Society of Anesthesiologists.

## Discussion

This study compared the performance of three measures of physical fitness, namely ASA grade, performance status, and the SCARF index, within an established model for case mix adjustment of 90-day mortality following CRC resection. The full model, incorporating all three measures of physical fitness, demonstrated the most accurate risk adjustment (pseudo-*R*^2^ = 0.193; C-statistic 0.835).

The SCARF index was the least predictive measure of physical fitness. Although excluding the SCARF index from the full model resulted in minimal reduction in model performance (pseudo-*R*^2^ = 99.6% of the full model; C-statistic 0.833), its limited contribution likely reflects several factors. First, there is substantial overlap of ICD-10 diagnostic codes between the RCS Charlson co-morbidity score and the SCARF index, introducing potential redundancy. Second, the SCARF index relies on accurate coding of patient diagnoses in administrative data sets, but co-morbidities within the SCARF index, such as pressure ulcers and urinary incontinence, have historically been poorly recorded^18^.

The SCARF index was originally developed using a 2-year look-back period from the date of breast cancer diagnosis. In the present study, the SCARF index was adapted by using a 2-year look-back period from the date of surgery, excluding ICD-10 codes in the surgical admission that could represent acute postoperative complications. This was to avoid misclassifying postoperative complications as deficits that contribute to the SCARF index. The SCARF index has been validated using 3-year overall survival, whereas the present study focused on short-term mortality. Despite the SCARF index’s established utility in other settings and its advantage of being derived from routinely collected administrative data, the near-identical performance of the more parsimonious model (ASA grade and performance status) compared with the full model, suggests that the inclusion of the SCARF index is not required for risk adjustment of postoperative mortality in patients with CRC. Alternative frailty measures can be derived from administrative data, including the Hospital Frailty Risk Score^45^ and the frailty syndromes model^46^. Compared with the SCARF index, these measures show similar model performance among patients with CRC^47^. In addition, the SCARF index was developed in a cohort of patients with cancer using HES and PEDW, whereas the Hospital Frailty Risk Score and frailty syndromes models were developed in older patients admitted as emergencies using only HES.

Unlike ASA grade and performance status, which require bespoke clinical data collection, the SCARF index is derived entirely from routinely collected administrative ICD-10 codes. Therefore, the SCARF index may represent the only available measure of physical fitness within some administrative data sets.

Of the individual measures of physical fitness, ASA grade was the most predictive and the SCARF index the least. The basic model plus performance status achieved similar discrimination and calibration to the basic model plus ASA grade. Measures of physical fitness based on clinician assessment (ASA grade and performance status) provided more accurate risk adjustment than the administratively derived SCARF index. This highlights the importance of clinician-reported measures of physical fitness for risk adjustment and supports their prioritization in data collection where feasible. This is reflected across international CRC registries. Registries including the Bowel Cancer Outcomes Registry^48^, NBOCA^29^, Dutch ColoRectal Audit^49^, Swedish Colorectal Cancer Registry^50^, and the American College of Surgeons cancer programmes^51^ capture and incorporate ASA grade within risk-adjustment models. Performance status is also captured in several registries, including the NBOCA, Danish Colorectal Cancer Group, and the American College of Surgeons cancer programmes^52^.

The utility of any measure of physical fitness is constrained by the completeness of its data across healthcare providers. In England, the Rapid Cancer Registration Data set (RCRD) became available for the first time in 2020. This routinely collected cancer registry data set has a reduced time lag of 3–6 months between patients being diagnosed with cancer and their data being available for analysis. National cancer audits use the RCRD to compare hospital performance in cancer care and outcomes, and support iterative quality-improvement cycles^53^. Case mix adjustment, including a measure of physical fitness, is required to ensure that these comparisons are accurate and fair, but a limitation of the RCRD is its data completeness. For example, among patients undergoing CRC resection between 1 July 2024 and 30 June 2025, data completeness for ASA grade was only 33%, with data completeness ranging from 6% to 100% between providers^20^.

Given the poor data completeness, ASA grade cannot be used for case mix adjustment in England using these data, even with strategies such as multiple imputation. In this scenario, the basic model plus performance status (83% data completeness in RCRD from 1 July 2024 to 30 June 2025) offers a viable alternative, given its comparable discriminative ability (C-statistic 0.822 for performance status *versus* 0.827 for ASA grade) and calibration (absolute difference between observed and predicted mortality < 0.5% across all deciles in both models). Unlike ASA grade, which is restricted to surgical patients, performance status is routinely recorded for all patients with cancer, providing an opportunity to extend the model to non-surgical populations.

A potential limitation of the present study is that 5.3% of patients recorded as undergoing a surgical resection in NBOCA had no corresponding record in HES or PEDW. For these patients, it was not possible to derive the Charlson score or SCARF index. Furthermore, 19.5% of records had missing data on at least one model covariate. To address missing data, missing data were imputed using multiple imputation with chained equations. Many of the variables included in the imputation model, such as ASA grade, performance status, age, sex, socioeconomic deprivation, surgical urgency, and postoperative mortality, are strongly predictive of co-morbidity burden. By including these variables in the imputation model, the ‘missing at random’ assumption is more plausible. Another potential limitation is that PEDW contains less diagnostic information than HES, with fewer diagnostic fields (14 *versus* 20). However, the SCARF index was developed using both HES and PEDW and has shown comparable performance between the two databases^18^.

The study period included the COVID-19 pandemic, which caused substantial disruption to care pathways^29,54^. To account for this, year of diagnosis was included in the risk-adjustment model. In addition, because models were developed and evaluated within the same cohort, estimates of model performance may be optimistic. However, relative differences between models would be expected to remain similar in external cohorts.

By comparing the performance of different measures of patient fitness, this study provides practical guidance for quality assurance programmes and observational studies to accurately adjust for physical fitness. This study demonstrated that performance status and ASA grade (measures based on clinical judgment) accurately adjust for physical fitness when risk adjusting postoperative mortality in patients with CRC, and outperform the SCARF index, an administrative frailty measure. When only one measure is available, performance status or ASA grade alone is suitable to support risk-adjusted hospital benchmarking.

## Supplementary Material

zrag130_Supplementary_Data

## Data Availability

The data that support the findings of this study are available from HQIP. Restrictions apply to the availability of these data, which were used under licence for this study. Access to data can be requested at https://www.hqip.org.uk/national-programmes/accessing-ncapop-data/#.Y-ZRBK3P1hE.
