# Supplementary material for: Measures of physical fitness and postoperative mortality in patients with colorectal cancer: national cohort study
Source: BJS Open. 2026 Oct 23;10(5):zrag130. doi: 10.1093/bjsopen/zrag130 (PMC13692692; doi:10.1093/bjsopen/zrag130)
Supplement: Supplementary file 1 [file zrag130_supplementary_data.docx]

**Measures of physical fitness and postoperative mortality in patients with colorectal cancer: a national cohort study**

Adil Rashid^1,2^, Helen A Blake^3^, Leo Watton^2,4,5^, Nicola Fearnhead^6^, Lu Han^1,2^, Jan van der Meulen^1,2^, Kate Walker^1,2^

^1^Department of Health Services Research and Policy, London School of Hygiene and Tropical Medicine, 15-17 Tavistock Place, London, WC1H 9SH, UK.

^2^Clinical Effectiveness Unit, Royal College of Surgeons of England, London, WC2A 3PE, UK.

^3^Research Department of Primary Care and Population Health, UCL Institute of Epidemiology and Health Care, 1-19 Torrington Place, London, WC1E 7HB, UK.

^4^Warrington and Halton NHS Trust, Warrington, WA5 1QG, UK.

^5^Institute of Science of Integrative Molecular Biology, University of Liverpool, L69 7ZB, UK.

^6^Department of Colorectal Surgery, Cambridge University Hospital NHS Foundation Trust, Cambridge, CB2 0QQ, UK.

**Corresponding author.** Adil Rashid, Department of Health Services Research and Policy, London School of Hygiene and Tropical Medicine, London, WC1H 9SH, UK (e-mail: [adil.rashid1@lshtm.ac.uk](mailto:adil.rashid1@lshtm.ac.uk)) **ORCID ID**; 0000-0003-2371-093X **Twitter** @Mr_AdilRashid

**Supplementary Materials - Index**

| **Supplementary Figures and Tables** |  |
| --- | --- |
| Supplementary Table 1: Surgical OPCS codes classified as surgical resections. | *pag. 2* |
| Supplementary Table 2: Data sources for all variables in the analysis. | *pag. 3* |
| Supplementary Table 3: Frequency of SCARF Index ICD-10 codes recorded in the HES/PEDW index surgical admission (N=87,027) and their classification as potential acute postoperative events. | *pag. 4* |
| Supplementary Table 4: Frequency of RCS Charlson Score ICD-10 codes recorded in the HES/PEDW index surgical admission (N=87,027) and their classification as potential acute postoperative events. | *pag. 6* |
| Supplementary Table 5: STROBE Statement—checklist of items that should be included in reports of observational studies. | *pag. 7* |
|  |  |

**Supplementary Figures and Tables**

Supplementary Table 1: Surgical OPCS codes classified as surgical resections.

| **Procedure group** | **OPCS code** | **Description** |
| --- | --- | --- |
| Right Hemi- colectomy | H06 | Extended excision of right hemicolon |
|  | H07 | Other excision of right hemicolon |
|  | H112 | Colectomy and side to side anastomosis of ileum to colon NEC |
|  | H116 | Colectomy and end to side anastomosis NEC |
|  | H118 | Other specified other excision of colon |
|  | H119 | Unspecified other excision of colon |
| Transverse colectomy | H08 | Excision of transverse colon |
| Left Hemi- colectomy | H09 | Excision of left hemicolon |
|  | H111 | Colectomy and end to end anastomosis of colon to colon NEC |
| Sigmoid colectomy | H10 | Excision of sigmoid colon |
| Total/Subtotal colectony | H04 | Total excision of colon and rectum |
|  | H05 | Total excision of colon |
|  | H29 | Subtotal excision of colon |
|  | H113 | Colectomy and anastomosis NEC |
|  | H114 | Colectomy and ileostomy NEC |
|  | H414 | Peranal mucosal proctectomy and endoanal anastomosis |
| Anterior Resection | H115 | Colectomy and exteriorisation of bowel NEC |
|  | H332 | Proctectomy and anastomosis of colon to anus |
|  | H333 | Anterior resection of rectum and anastomosis of colon to rectum using staples |
|  | H334 | Anterior resection of rectum and anastomosis NEC |
|  | H336 | Anterior resection of rectum and exteriorisation of bowel |
|  | H338 | Other specified excision of rectum |
|  | H339 | Unspecified excision of rectum |
|  | H404 | Trans-sphincteric anastomosis of colon to anus |
|  | H411 | Rectosigmoidectomy and peranal anastomosis |
| Hartmanns | H335 | Rectosigmoidectomy and closure of rectal stump and exteriorisation of bowel |
| Abdomino Perineal Excision of Rectum | H331 | Abdominoperineal excision of rectum and end colostomy |
|  | H337 | Perineal resection of rectum HFQ |
| Pelvic Exenteration | X14 | Clearance of pelvis |

Supplementary Table 2: Data sources for all variables in the analysis.

| **Source of data items** | **NBOCA** | **HES/PEDW** | **ONS** |
| --- | --- | --- | --- |
| **Exposure:** |  |  |  |
| Surgical resection | ✓ |  |  |
| **Outcome:** |  |  |  |
| 90-day mortality |  |  | ✓ |
| **Patient and tumour characteristics:** |  |  |  |
| Calendar period | ✓ |  |  |
| Sex | ✓^1^ | ✓^2^ |  |
| Age | ✓ |  |  |
| ASA grade | ✓ |  |  |
| TNM stage | ✓ |  |  |
| Cancer site | ✓ |  |  |
| Comorbidities |  | ✓ |  |
| SCARF index |  | ✓ |  |
| Performance Status | ✓ |  |  |
| Mode of admission | ✓^2^ | ✓^1^ |  |

Where data was available from more than one source, order of preference for reconciliation rules are given using numbers in superscript.

NBOCA, National Bowel Cancer Audit.

HES, Hospital Episode Statistics.

PEDW, Patient Episode Database for Wales.

ONS, Office of National Statistics.

Supplementary Table 3: Frequency of SCARF Index ICD-10 codes recorded in the HES/PEDW index surgical admission (N=87,027) and their classification as potential acute postoperative events.

| **ICD-10 code and description** | **Frequency** | **Percentage** | **Classified as acute postoperative event†** |
| --- | --- | --- | --- |
| Y83 Surgical operation as the cause of abnormal reaction of the patient, without mention of misadventure at the time of procedure | 11,874 | 13.64% | Yes |
| R40 Somnolence, stupor and coma | 94 | 0.11% | Yes |
| W19 Unspecified fall | 108 | 0.12% | Yes |
| L03 Cellulitis and acute lymphangitis | 966 | 1.11% | Yes |
| L89 Pressure ulcer | 645 | 0.74% | Yes |
| E87 Other fluid/electrolyte/acid-base disorders | 5,316 | 6.11% | Yes |
| R63 Symptoms concerning food/fluid intake | 408 | 0.47% | Yes |
| F05 Delirium | 1,432 | 1.65% | Yes |
| I44 AV/bundle-branch block | 1,308 | 1.50% | No |
| I48 Atrial fibrillation/flutter | 9,373 | 10.77% | Yes |
| I49 Other cardiac arrhythmias | 426 | 0.49% | Yes |
| I63 Cerebral infarction | 111 | 0.13% | Yes |
| I65 Occlusion and stenosis of precerebral arteries, not resulting in cerebral infarction | 96 | 0.11% | No |
| N19 Unspecified kidney failure | 126 | 0.14% | Yes |
| I95 Hypotension | 3,755 | 4.31% | Yes |
| R55 Syncope and collapse | 480 | 0.55% | Yes |
| R42 Dizziness and giddiness | 351 | 0.40% | Yes |
| E86 Volume depletion | 1,469 | 1.69% | Yes |
| I20 Angina pectoris | 2,934 | 3.37% | No |
| I21 Acute myocardial infarction | 226 | 0.26% | Yes |
| K559 Vascular disorder of intestine, unspecified | 282 | 0.32% | Yes |
| R02 Gangrene, not elsewhere classified | 270 | 0.31% | Yes |
| J15 Bacterial pneumonia, not elsewhere classified | 93 | 0.11% | Yes |
| J18 Pneumonia, unspecified organism | 3,984 | 4.58% | Yes |
| J22 Unspecified acute lower respiratory infection | 530 | 0.61% | Yes |
| J90 Pleural effusion, not elsewhere classified | 3,408 | 3.92% | Yes |
| R06 Abnormalities of breathing | 484 | 0.56% | Yes |
| R31 Unspecified hematuria | 448 | 0.51% | Yes |
| R33 Retention of urine | 3,561 | 4.09% | Yes |
| N390 Urinary tract infection, site not specified | 2,131 | 2.45% | Yes |

†Codes classified as acute were excluded from the SCARF Index when recorded during the index surgical admission.

The following ICD-10 codes, J46 (Status asthmaticus), I22 (Subsequent myocardial infarction), and I23 (Complications following acute MI), were already classified as acute in the RCS Charlson score and were therefore also classified as acute in the SCARF index.

Rare ICD-10 codes (fewer than 0.01% in the index admissions) are not reported.

Supplementary Table 4: Frequency of RCS Charlson Score ICD-10 codes recorded in the HES/PEDW index surgical admission (N=87,027) and their classification as potential acute postoperative events.

| **ICD-10 code and description** | **Frequency** | **Percentage** | **Classified as acute postoperative event†** |
| --- | --- | --- | --- |
| I26 Pulmonary embolism | 640 | 0.74% | Yes |
| K559 Vascular disorder of intestine, unspecified | 282 | 0.32% | Yes |
| R02 Gangrene, not elsewhere classified | 270 | 0.31% | Yes |
| I63 Cerebral infarction | 111 | 0.13% | Yes |
| I65 Occlusion and stenosis of precerebral arteries, not resulting in cerebral infarction | 96 | 0.11% | No |

†Codes classified as acute were excluded from the RCS Charlson score when recorded during the index surgical admission.

Rare ICD-10 codes (fewer than 0.01% in the index admissions) are not reported.

Supplementary Table 5: STROBE Statement—checklist of items that should be included in reports of observational studies.

|  | Item No | Recommendation | Reported on Page No |
| --- | --- | --- | --- |
| **Title and abstract** | 1 | (*a*) Indicate the study’s design with a commonly used term in the title or the abstract | 1 |
|  |  | (*b*) Provide in the abstract an informative and balanced summary of what was done and what was found | 2 |
| Introduction | | |  |
| Background/rationale | 2 | Explain the scientific background and rationale for the investigation being reported | 3 |
| Objectives | 3 | State specific objectives, including any prespecified hypotheses | 4 |
| Methods | | |  |
| Study design | 4 | Present key elements of study design early in the paper | 5-7 |
| Setting | 5 | Describe the setting, locations, and relevant dates, including periods of recruitment, exposure, follow-up, and data collection | 5 |
| Participants | 6 | (*a*) *Cohort study*—Give the eligibility criteria, and the sources and methods of selection of participants. Describe methods of follow-up | 5 |
|  |  | (*b*) *Cohort study*—For matched studies, give matching criteria and number of exposed and unexposed | N/A |
| Variables | 7 | Clearly define all outcomes, exposures, predictors, potential confounders, and effect modifiers. Give diagnostic criteria, if applicable | 5-7 |
| Data sources/ measurement | 8* | For each variable of interest, give sources of data and details of methods of assessment (measurement). Describe comparability of assessment methods if there is more than one group | Supplementary Table 2 |
| Bias | 9 | Describe any efforts to address potential sources of bias | 6 (Handling missing data) |
| Study size | 10 | Explain how the study size was arrived at | Figure 1 |
| Quantitative variables | 11 | Explain how quantitative variables were handled in the analyses. If applicable, describe which groupings were chosen and why | 5-7 |
| Statistical methods | 12 | (*a*) Describe all statistical methods, including those used to control for confounding | 6-7 |
|  |  | (*b*) Describe any methods used to examine subgroups and interactions | N/A |
|  |  | (*c*) Explain how missing data were addressed | 6 |
|  |  | (*d*) *Cohort study*—If applicable, explain how loss to follow-up was addressed | N/A |
|  |  | (*e*) Describe any sensitivity analyses | N/A |

| Results | | |  |
| --- | --- | --- | --- |
| Participants | 13* | (a) Report numbers of individuals at each stage of study—eg numbers potentially eligible, examined for eligibility, confirmed eligible, included in the study, completing follow-up, and analysed | Figure 1 |
|  |  | (b) Give reasons for non-participation at each stage | Figure 1 |
|  |  | (c) Consider use of a flow diagram | Figure 1 |
| Descriptive data | 14* | (a) Give characteristics of study participants (eg demographic, clinical, social) and information on exposures and potential confounders | Table 2 |
|  |  | (b) Indicate number of participants with missing data for each variable of interest | Table 2 |
|  |  | (c) *Cohort study*—Summarise follow-up time (eg, average and total amount) | 6 |
| Outcome data | 15* | *Cohort study*—Report numbers of outcome events or summary measures over time | Table 2 |
| Main results | 16 | (*a*) Give unadjusted estimates and, if applicable, confounder-adjusted estimates and their precision (eg, 95% confidence interval). Make clear which confounders were adjusted for and why they were included | 7-9  Table 3, 4 |
|  |  | (*b*) Report category boundaries when continuous variables were categorized | Table 3 |
|  |  | (*c*) If relevant, consider translating estimates of relative risk into absolute risk for a meaningful time period | N/A |
| Other analyses | 17 | Report other analyses done—eg analyses of subgroups and interactions, and sensitivity analyses | N/A |
| Discussion | | |  |
| Key results | 18 | Summarise key results with reference to study objectives | 10-12 |
| Limitations | 19 | Discuss limitations of the study, taking into account sources of potential bias or imprecision. Discuss both direction and magnitude of any potential bias | 10-12 |
| Interpretation | 20 | Give a cautious overall interpretation of results considering objectives, limitations, multiplicity of analyses, results from similar studies, and other relevant evidence | 10-12 |
| Generalisability | 21 | Discuss the generalisability (external validity) of the study results | 10-12 |
| Other information | | |  |
| Funding | 22 | Give the source of funding and the role of the funders for the present study and, if applicable, for the original study on which the present article is based | 1 |

*Give information separately for cases and controls in case-control studies and, if applicable, for exposed and unexposed groups in cohort and cross-sectional studies.

**Note:** An Explanation and Elaboration article discusses each checklist item and gives methodological background and published examples of transparent reporting. The STROBE checklist is best used in conjunction with this article (freely available on the Web sites of PLoS Medicine at http://www.plosmedicine.org/, Annals of Internal Medicine at http://www.annals.org/, and Epidemiology at http://www.epidem.com/). Information on the STROBE Initiative is available at www.strobe-statement.org.
